# Hemagglutinin virus-like particles incorporated with membrane-bound cytokine adjuvants provide protection against homologous and heterologous influenza virus challenge in aged mice

**DOI:** 10.1186/s12979-023-00344-w

**Published:** 2023-05-11

**Authors:** Bo Ryoung Park, Ramireddy Bommireddy, David Hyunjung Chung, Ki-Hye Kim, Jeeva Subbiah, Yu-Jin Jung, Noopur Bhatnagar, Christopher D. Pack, Sampath Ramachandiran, Shaker J.C. Reddy, Periasamy Selvaraj, Sang-Moo Kang

**Affiliations:** 1 Institute for Biomedical Sciences, Georgia State University, Atlanta, GA 30303 USA; 2 Department of Pathology and Laboratory Medicine, Emory University School of Medicine, Atlanta, GA 30322 USA; 3 Metaclipse Therapeutics Corporation, Atlanta, GA 30340 USA

**Keywords:** Influenza, Virus-like particles (HA-VLPs), Hemagglutinin (HA), Cross protection, Cytokine adjuvants, GPI-IL-12, GPI-GM-CSF, HA-VLP, Aged mice, T cells

## Abstract

**Background:**

Current influenza vaccines deliver satisfactory results in young people but are less effective in the elderly. Development of vaccines for an ever-increasing aging population has been an arduous challenge due to immunosenescence that impairs the immune response in the aged, both quantitatively and qualitatively.

**Results:**

To potentially enhance vaccine efficacy in the elderly, we investigated the immunogenicity and cross-protection of influenza hemagglutinin virus-like particles (HA-VLP) incorporated with glycosylphosphatidylinositol (GPI)-anchored cytokine-adjuvants (GPI-GM-CSF and GPI-IL-12) via protein transfer in aged mice. Lung viral replication against homologous and heterologous influenza viruses was significantly reduced in aged mice after vaccination with cytokine incorporated VLPs (HA-VLP-Cyt) in comparison to HA-VLP alone. Enhanced IFN-γ^+^CD4^+^ and IFN-γ^+^CD8^+^ T cell responses were also observed in aged mice immunized with HA-VLP-Cyt when compared to HA-VLP alone.

**Conclusions:**

Cytokine-adjuvanted influenza HA-VLP vaccine induced enhanced protective response against homologous influenza A virus infection in aged mice. Influenza HA-VLP vaccine with GPI-cytokines also induced enhanced T cell responses correlating with better protection against heterologous infection in the absence of neutralizing antibodies. The results suggest that a vaccination strategy using cytokine-adjuvanted influenza HA-VLPs could be used to enhance protection against influenza A virus in the elderly.

## Introduction

Influenza viruses belong to the Orthomyxoviridae family and consist of eight segmented negative-sense single-stranded RNAs [1]. Highly transmissible influenza A viruses often induce human respiratory illness and such infection can lead to outbreaks or pandemics [2]. CDC estimates that 70–85% of seasonal flu-related deaths occur among people 65 years and older. Although vaccination with inactivated viruses has been proven to be an effective measure to prevent influenza virus infection, vaccine effectiveness is suboptimum in the elderly population compared to young adults [3], 17–53% compared to 70–90% in young adults [4, 5]. This phenomenon of low responses to vaccination is mainly linked to the weakened immune system and partially due to original antigenic sin [4, 6, 7]. Seroconversion rates post vaccination for the elderly are less than 30%, while young adults demonstrate 50–75% seroconversion rates [8]. The decreased vaccine-induced humoral responses specific for vaccine strains can be partially attributed to the reduction of both neutralizing and Hemagglutination inhibition (HAI) activity of antibodies in elderly individuals [9]. Recent approaches to improve vaccine efficacy in the elderly include high-dose vaccines [10, 11] and the use of potent adjuvants [12]. Currently, a high dose influenza vaccine and an adjuvanted vaccine are approved for use in the US in people 65 years and older [13]. These influenza vaccines targeted to the elderly induce elevated HAI titers, resulting in enhanced efficacy of the vaccines [14–16]. However, the long-term consequences of repeated administration of large doses of vaccine adjuvants is not fully known warranting further investigation of potentially safer, immune cell targeted vaccine adjuvants that can be used in elderly population.

Virus-like particles (VLPs) are self-assembling nanoparticles (20–200 nm) that closely resemble the structure of the virus in size and shape but lack viral genomes [17]. The nanosized VLPs traffic through lymph nodes and are taken up by antigen-presenting cells (APCs) [18]. VLPs display diverse antigenic epitopes and induce both humoral and cellular anti-viral immune responses [19]. Vaccination with VLPs containing hemagglutinin (HA) from influenza A/California/2009 was shown to induce a more protective immune response in ferrets in comparison to a conventional inactivated virus vaccination [20]. Another advantage of VLPs is that it can be modified to display immunostimulatory cytokines as adjuvants to enhance the vaccine efficacy [21, 22]. These features suggest that VLPs can be used as a potent vaccine platform to develop vaccines for influenza.

Endogenous immune activators such as granulocyte macrophage colony-stimulating factor (GM-CSF) and interleukin-12 (IL-12) have been reported to enhance the immunogenicity of vaccines for Ebola, SARS-CoV-2 and influenza viruses [22–26]. To circumvent ineffective immune responses in the aged, we engineered  glycosylphosphatidylinositol (GPI)-anchored form of cytokines which can be incorporated into influenza HA-VLPs by a simple protein transfer technique developed in our laboratory. The glycolipid anchor permits the incorporation of purified GPI-anchored proteins into the lipid bilayer of influenza HA-VLPs [22]. Using this technique, we modified influenza HA-VLPs with these biological adjuvants (immunostimulatory cytokines) to augment vaccine uptake, enhance antigen presentation, and activate APCs such as dendritic cells (DCs) [27]. By introducing the membrane incorporated GPI-cytokines into HA-VLPs, multiple antigens can more effectively be presented to the immune system in a targeted manner, resulting in a more robust immune response. In addition, administration of HA-VLP vaccines containing membrane-anchored cytokines can localize the cytokines to the area of injection, thereby reducing the toxic effects associated with systemically administered soluble cytokines. Moreover, the physical linkage of adjuvant and antigen source can present the adjuvant and antigen to the immune cells simultaneously, resulting in enhanced vaccine efficacy. This combination approach is more effective and efficient than unconjugated antigen and adjuvant mixtures [28, 29].

Our earlier studies have shown that incorporation of GPI-anchored GM-CSF into the HA-VLP lipid envelope improves anti-viral immunity after vaccination of young adult mice [21, 22]. Here, influenza VLP vaccines that express HA from influenza A/PR8 virus (HA-VLP) were modified to display GPI-GM-CSF and GPI-IL-12 on the HA-VLP surface via protein transfer. The goal of this study was to determine whether membrane cytokine incorporated influenza HA-VLP vaccines enhances protective humoral and cellular immunity in aged mice and compare resulting immune responses to unmodified HA-VLPs. PR8 HA VLP displaying cytokines (HA-VLP-Cyt) conferred enhanced protection against homologous and heterologous influenza viruses and reduced lung viral titers in aged mice in comparison to unmodified HA-VLP. The results from this study support a potential strategy of improving protection in the elderly by enhancing cellular and humoral immunity through the use of influenza HA-VLPs modified with GPI-GM-CSF and GPI-IL-12 immune activators.

## Results

### Vaccine Preparation by protein transfer of GPI-GM-CSF and GPI-IL-12 onto HA-VLP

**Fig. 1:**
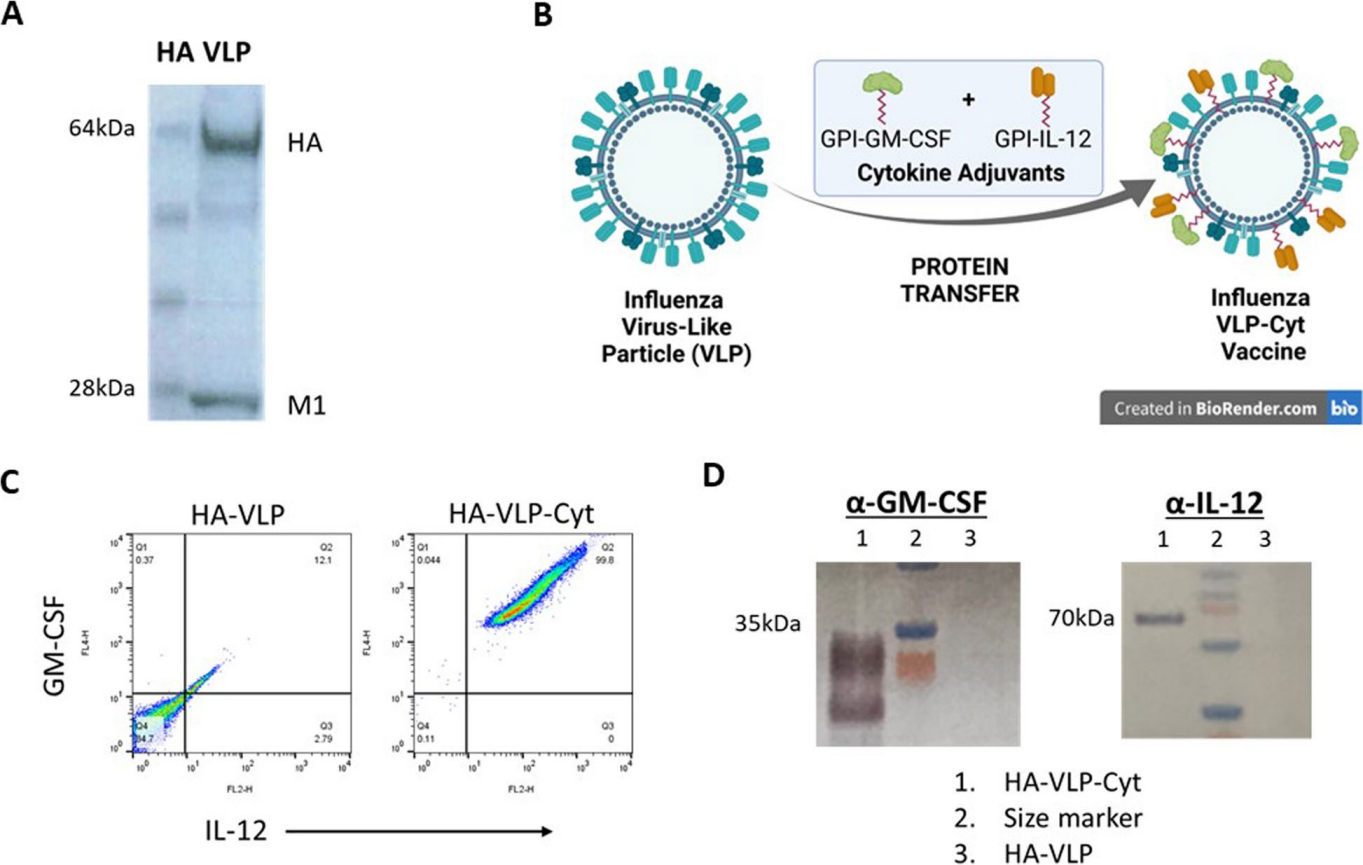
Development and characterization of influenza A/PR8 HA-VLP and A/PR8 HA VLP-cytokines modified VLP vaccine. **A** SDS-PAGE gel colloidal blue staining of PR8 HA-VLP expressed in insect cells after purification, displaying HA and M1 proteins. **B** GPI-cytokines incorporation schema for HA-VLPs (created with Biorender.com). **C** Flow cytometry analysis of HA-VLP (control) and HA-VLP-Cytokines vaccine incorporated GPI-GM-CSF and GPI-IL-12 (HA-VLP-Cyt). **D** Western blot analysis of incorporated GPI-GM-CSF or GPI-IL-12 in HA-VLPs using anti-mouse GM-CSF antibody and anti-mouse IL-12 p40 antibody respectively. PR8 HA-VLP contains HA from A/PR8/34, PR8 HA-VLP-Cyt contains HA and cytokines (GM-CSF and IL-12). Size markers are shown only for the region of interest

Influenza HA-VLPs containing codon-optimized hemagglutinin (H1 HA) and matrix M1 proteins derived from A/Puerto Rico/8/1934 (PR8) were produced in insect cells (Sf9) as described [30] and purified by tangential flow diafiltration and anion exchange (Capto Q) chromatography by Medigen (Frederick, MD, USA). Influenza HA-VLPs were characterized by SDS-PAGE and Coomassie blue staining and shown to exhibit a high purity of major influenza virus protein components HA and M1 (Fig. 1A). We incorporated affinity purified GPI-GM-CSF and GPI-IL-12 into HA-VLPs by protein transfer to prepare our VLP-GM-CSF-IL-12 (HA-VLP-Cyt) vaccine as described (Fig. 1B) [21]. GPI-cytokine incorporation into HA-VLP was detected by flow cytometry analysis (Fig. 1C) and confirmed by Western blot using anti-mouse GM-CSF and anti-mouse IL-12 antibodies (Fig. 1D). Primary antibodies were revealed using a Goat anti-rat AP secondary antibody.

### Cytokines enhance VLP vaccine-induced antibody response in aged mice

**Fig. 2:**
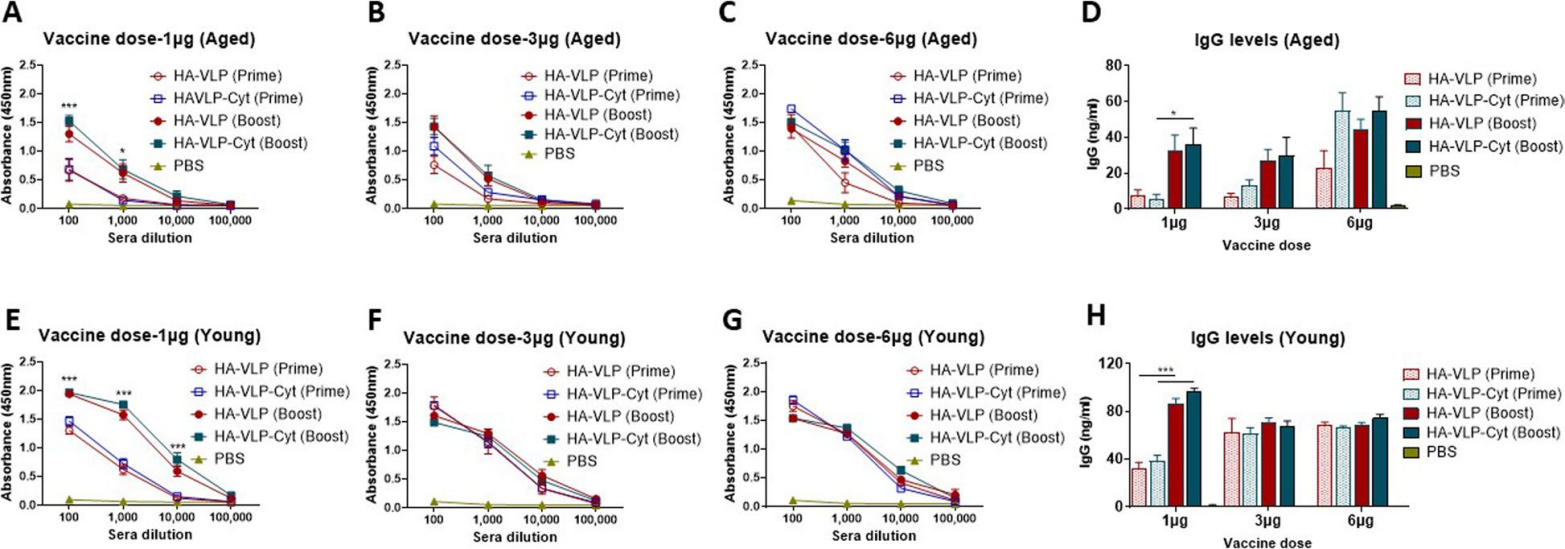
Induction of IgG antibodies by HA-VLP and HA-VLP-Cyt vaccines. Aged (**A**-**D**) and young (**E**-**H**) BALB/c mice (*n=* 6/group) were immunized with 1 µg (**A**, **E**), 3 µg (**B**, **F**) or 6 µg (**C**, **G**) of HA-VLP or HA-VLP-Cyt. Mouse sera were collected at 2 weeks after prime or boost immunization. (**D**, **H**) IgG antibody responses to A/PR8 virus were measured in prime or boost immune sera and quantified in 1:1,000 sera. Error bars indicate mean ± SEM. * *p*<0.05; *** *p*<0.001

Herein we investigated the immunogenicity and efficacy of A/PR8/1934 (H1N1) VLP (HA-VLP) and HA-VLP incorporated with GPI-GM-CSF and GPI-IL-12 (HA-VLP-Cyt) in young and aged (16–18 months old) BALB/c mice. The mice were immunized intramuscularly with HA-VLP or HA-VLP-Cyt with escalating doses of vaccine (1, 3 or 6 µg, Fig. 2). Although aged mice responded to HA-VLP and HA-VLP-Cyt similarly at the high dose (6 µg, Fig. 2C), the cytokine adjuvant increased the antibody response at a low dose (1 µg) compared to HA-VLP (Fig. 2A). However, vaccination of young adult mice with HA-VLP and HA-VLP-Cyt induced similar levels of A/PR8 virus specific IgG antibody response (Fig. 2E). However, they require a booster dose at the low dose (1 µg, Fig. 2E) but not at higher doses (3 and 6 µg, Fig. 2F and G). When increasing the HA-VLP and HA-VLP-Cyt vaccine to a moderate (3 µg, Fig. 2B) or high dose (6 µg, Fig. 2C) level, the aged mice produced substantially higher levels of IgG antibodies specific for A/PR8 virus with a significant increase in IgG production by HA-VLP-Cyt compared to HA-VLP. The effects of the booster dose on enhancing IgG antibody responses were most prominent at the low dose (1 µg) of HA-VLP or HA-VLP-Cyt primed young (Fig. 2E and H) and aged mice (Fig. 2A and D). Booster immunization in aged mice with 3 or 6 µg of HA-VLP (Fig. 2B and C) resulted in a moderate increase in IgG levels specific for A/PR8 virus, but no difference was observed in the mice that received a high dose (6 µg) of HA-VLP-Cyt vaccine (Fig. 2C and D). Booster dose with 3 or 6 µg of HA-VLP or HA-VLP-Cyt did not further increase the prime-induced levels of IgG antibodies to A/PR8 virus in young adult mice (Fig. 2F, G and H).

**Fig. 3:**
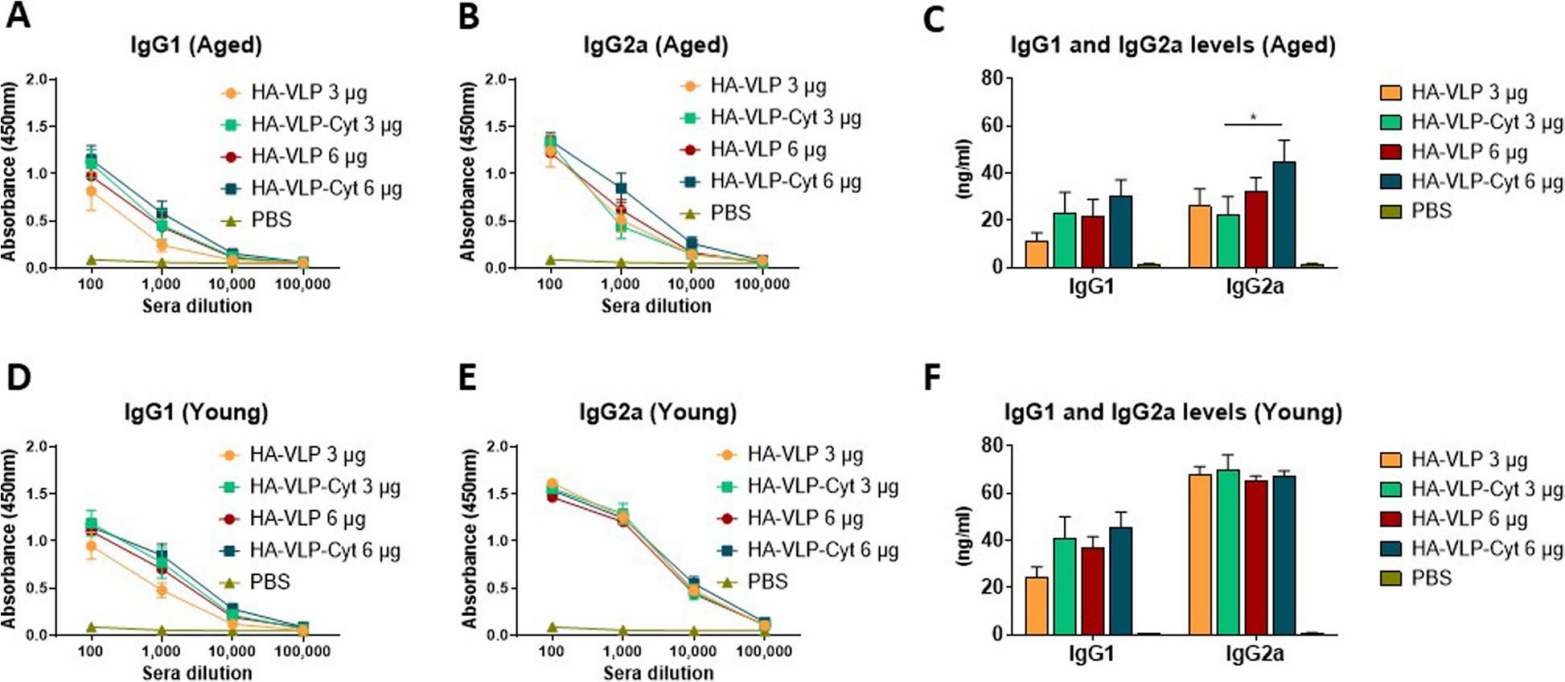
HA-VLP-Cyt vaccine induces A/PR8 specific IgG2a antibody responses at a higher dose in aged mice. Aged (**A**-**C**) and young (**D**-**F**) BALB/c mice (*n=* 6/group) received boost dose of HA-VLP or HA-VLP-Cyt. IgG1 (**A**, **D**) and IgG2a (**B**, **E**) antibody responses to A/PR8 2 weeks after 3 µg or 6 µg of HA-VLP or HA-VLP-Cyt inoculation. (**C**, **F**) A/PR8 specific IgG1 and IgG2a antibodies levels were quantified in 1:1,000 boost sera. Error bars indicate mean ± SEM. * *p*<0.05. 3 µg HA-VLP-Cyt and 6 µg HA-VLP-Cyt groups were compared

To determine whether the HA-VLP-Cyt induces a predominant Th1 or Th2 type humoral response, immunoglobulin isotypes IgG1 (Th2 type) and IgG2a (Th1 type) levels were determined after booster dose. Moderate to high doses of HA-VLP-Cyt (3 or 6 µg) immunization induced slightly increased IgG1 antibody responses to A/PR8 compared to HA-VLP vaccination in both aged (Fig. 3A and C) and young mice (Fig. 3D and F). However, the high dose of HA-VLP-Cyt induced a significant increase in IgG2a levels in aged mice (Fig. 3B and C) while no additional benefit of the cytokines was observed in young adult mice (Fig. 3E and F). These results suggest that HA-VLP and HA-VLP-Cyt effectively primed the induction of A/PR8 specific IgG antibodies in aged mice in a dose dependent manner. The ability of low dose HA-VLP or HA-VLP-Cyt prime and boost vaccination to induce IgG antibodies was significantly compromised in aged mice compared to young adult mice. However, increasing the HA-VLP-Cyt dose resulted in an enhanced IgG2a antibody response in aged mice.

### HA-VLP-Cyt vaccine induces increased levels of cross-reactive IgG antibody responses

**Fig. 4:**
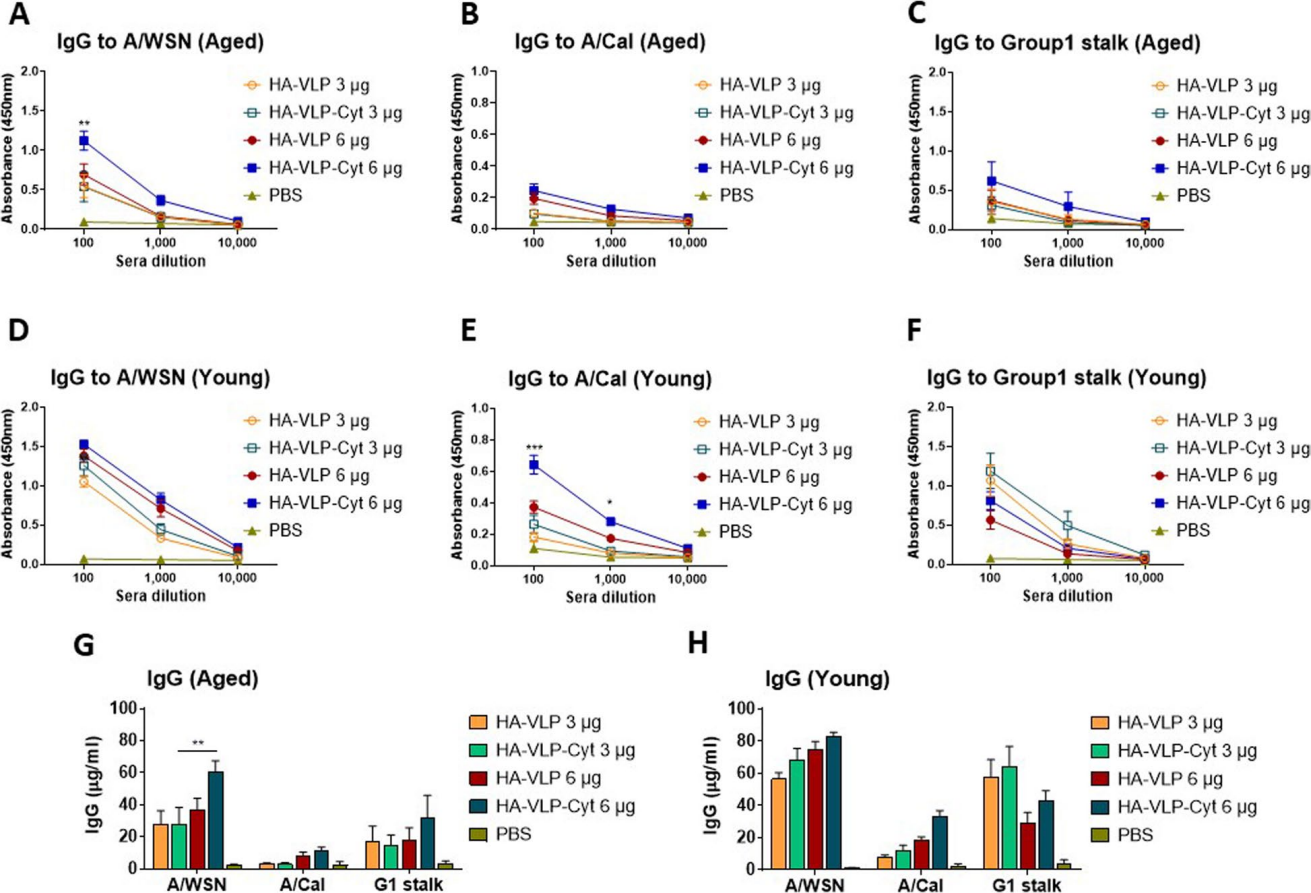
HA-VLP or HA-VLP-Cyt vaccination-induced antibody recognizes heterologous A/WSN, A/Cal and group 1 stalk domain. Immune sera were collected at 2 weeks after boost immunization with HA-VLP or HA-VLP-Cyt from young adult and aged BALB/c mice (*n=* 6/group). HA-VLP or HA-VLP-Cyt with vaccine dose 3 µg or 6 µg were inoculated intramuscularly in two doses at an interval of 3 weeks. IgG antibody responses to A/WSN (**A**, **D**), A/Cal (**B**, **E**), and group 1 stalk domain (**C**, **F**) were measured and quantified in 1:100 boost sera (**G**, **H**). Error bars indicate mean ± SEM. * *p*<0.05; ** *p*<0.01; *** *p*<0.001

To determine whether cytokine adjuvants enhance the generation of cross-reactive antibodies, sera after boost immunization with HA-VLP or HA-VLP-Cyt were analyzed for cross reactivity against heterologous viruses, A/WSN/1933 (H1N1) and A/California/2009 (H1N1), in young adult and aged mice. Boost sera from the aged mice displayed significantly lower levels (~ 3 fold) of IgG antibodies specific for A/California/2009 (H1N1) compared to A/WSN/1933 (H1N1) (Fig. 4A and B). With a higher dose (6 µg) of HA-VLP or HA-VLP-Cyt, boost sera from young adult (Fig. 4D and H) and aged mice (Fig. 4A and G) showed increased levels of IgG antibody reactivity to A/WSN than with 3 µg dose. We observed a similar pattern of cross-reactive IgG antibodies recognizing A/California/2009 (H1N1) (Fig. 4B and E) but at 3- to 6-fold lower levels than those recognizing A/WSN in both young adult and aged mice, most likely due to antigenic distance (Fig. 4B and E compared to Fig. 4A and D). However, HA-VLP-Cyt induced a higher titer of IgG against A/California/2009 in young adult mice (Fig. 4E) than HA-VLP, suggesting that cytokine adjuvants present in the HA-VLP vaccine increase the antibody response against a heterologous influenza A strain. Higher IgG antibodies to group 1 HA stalk were induced in young adult mice (Fig. 4F) than aged mice (Fig. 4C) after boost dose of HA-VLP or HA-VLP-Cyt. These results suggest that aged mice have a reduced capacity to induce cross reactive IgG antibody responses to heterologous viruses compared to young adult mice. However, addition of cytokine adjuvants (HA-VLP-Cyt) as well as a higher vaccine dose improves the cross-reactive antibodies against group 1 stalk in aged mice (Fig. 4G).

### Aged mice require a higher dose of HA-VLP and HA-VLP-Cyt to induce comparable levels of hemagglutination inhibition (HAI) titers

**Fig. 5:**
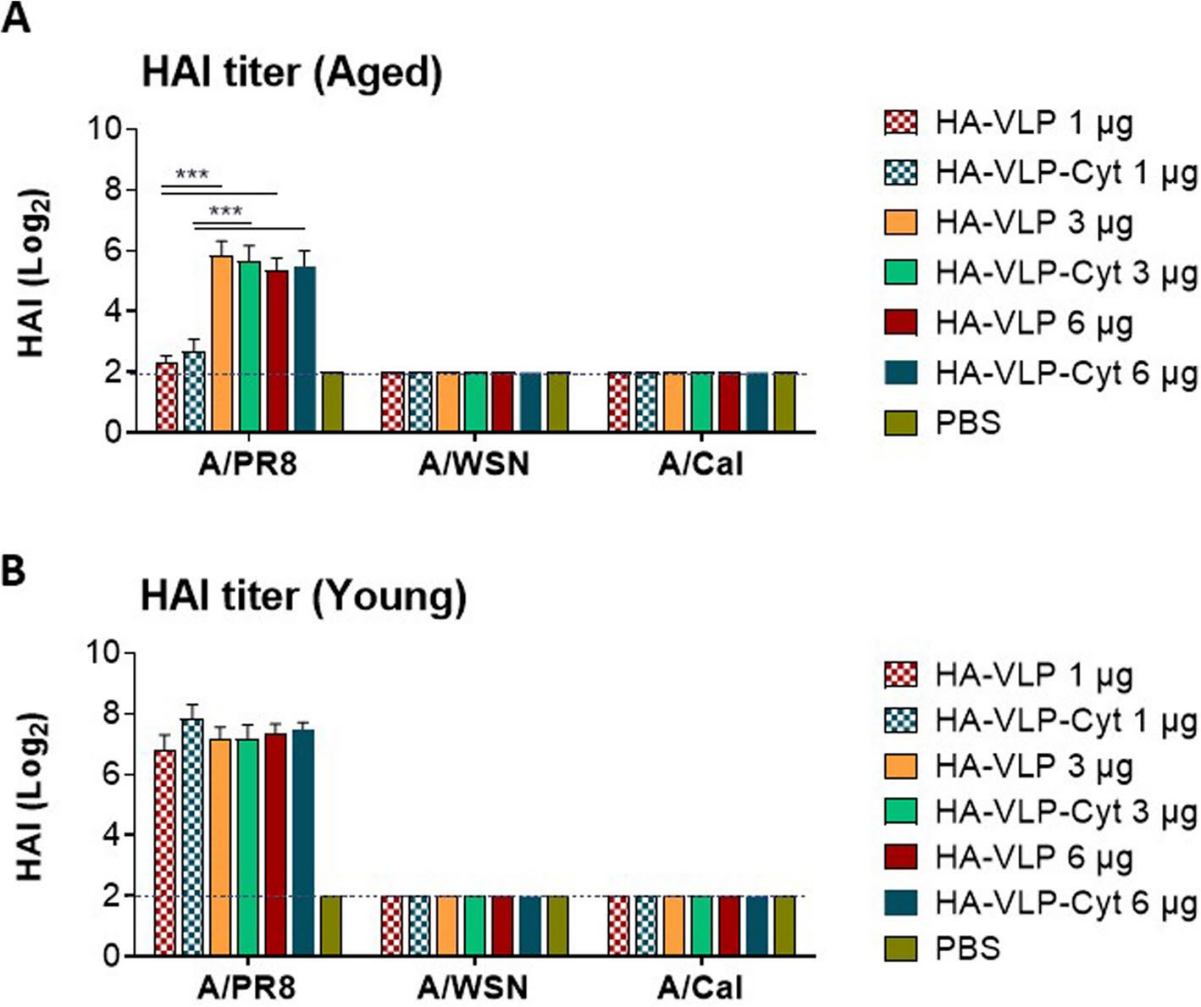
HA-VLP and HA-VLP-Cyt vaccine-induced antibody inhibits hemagglutination of homologous but not heterologous viruses. HAI titers against the viruses (A/PR8, A/WSN, and A/Cal) were determined in sera from aged (**A**) and young (**B**) mice (*n=* 6/group) at 2 weeks after boost immunization with HA-VLP or HA-VLP-Cyt. 1 µg, 3 µg or 6 µg of each HA-VLP and HA-VLP-Cyt were administered. The statistical significances were determined using two-way ANOVA and indicated in *** *P <* 0.001. Error bars indicate mean ± SEM

To determine hemagglutination inhibition (HAI) titers, sera from young adult and aged mice were collected after a booster immunization with 1, 3 or 6 µg of HA-VLP or HA-VLP-Cyt. HAI activity was observed in all doses of immune sera against A/PR8 but not against heterologous viruses A/WSN and A/Cal (Fig. 5). Although both young and old age groups with HA-VLP or HA-VLP-Cyt immunization showed significantly increased HAI titers than the control group, aged mice (Fig. 5A) required a 3-fold higher HA-VLP or HA-VLP-Cyt dose than young adult mice (Fig. 5B) to induce comparable levels of HAI titers. Compared to the 1 µg dose, increased doses of immunization (3 or 6 µg) resulted in 8-16-fold elevated HAI titers in aged mice (Fig. 5A). These results suggest that a higher vaccine dose is required for aged mice.

### An increased dose of HA-VLP and HA-VLP-Cyt vaccination is required for protection in aged mice

**Fig. 6:**
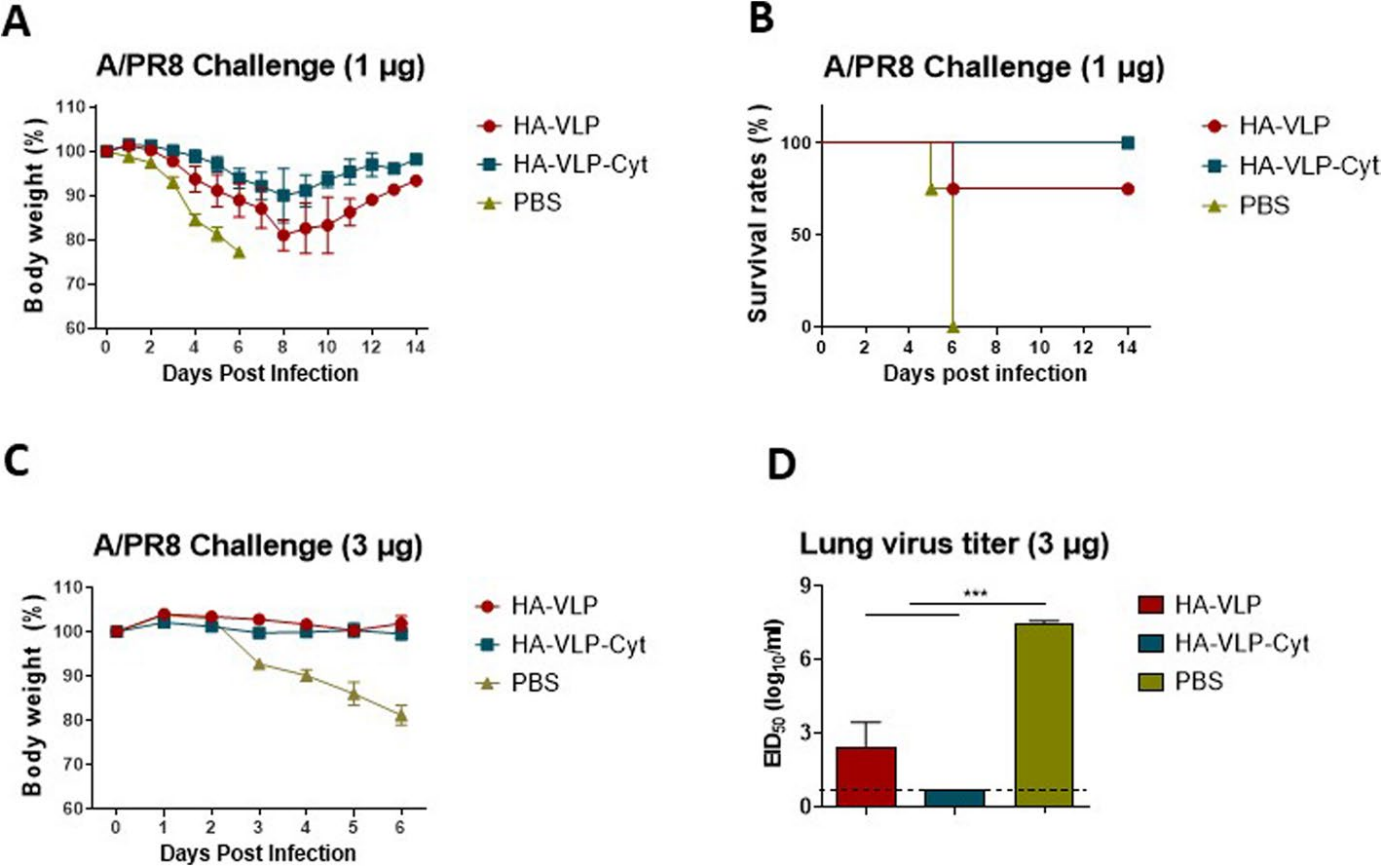
GPI-cytokine incorporation into HA-VLP vaccine reduces lung virus load and enhances survival of aged mice. Body weight changes (**A** and **C**), survival (**B**) and lung virus titer (**D**) at day 6 after A/PR8 challenge. Aged BALB/c mice (*n=* 5/group) were prime-boost immunized with 1 µg (**A**, **B**) or 3 µg (**C**, **D**) of HA-VLP or HA-VLP-Cyt prior to A/PR8 virus infection (2 LD_50_, 103.36 EID_50_). The statistical significances were determined using One-way ANOVA and Turkey's multiple comparison test and indicated in *** *P <* 0.001. Error bars indicate mean ± SEM

To determine protective efficacy, aged BALB/c mice were immunized with low dose HA-VLP or HA-VLP-Cyt (1 µg) and then challenged with a lethal dose of A/PR8 after 4 weeks. The aged mouse group receiving a low dose of HA-VLP vaccination showed severe weight loss (~ 20%) with 80% survival rates, whereas the HA-VLP-Cyt vaccinated group lost less body weight (~ 10%) and had 100% survival. Unvaccinated mice did not survive after infection (Fig. 6A and B). No body weight loss was observed in both HA-VLP and HA-VLP-Cyt groups receiving the 3-fold higher dose (3 µg) of HA-VLP or HA-VLP-Cyt after challenge with a lethal dose of A/PR8 (Fig. 6C). HA-VLP vaccination displayed lower lung virus titers (2.4 log_10_EID_50_/ml, Fig. 6D) compared to the unvaccinated group showing 100,000 folds higher titers (7.4 log_10_EID_50_/ml). Notably, no virus was detected in the lung of HA-VLP-Cyt aged mice group (Fig. 6D). These results suggest that HA-VLP-Cyt improves protection by effectively controlling viral replication in the lung.

### Aged mice require a higher dose of HA-VLP or HA-VLP-Cyt vaccine to induce heterologous cross-protection

**Fig. 7:**
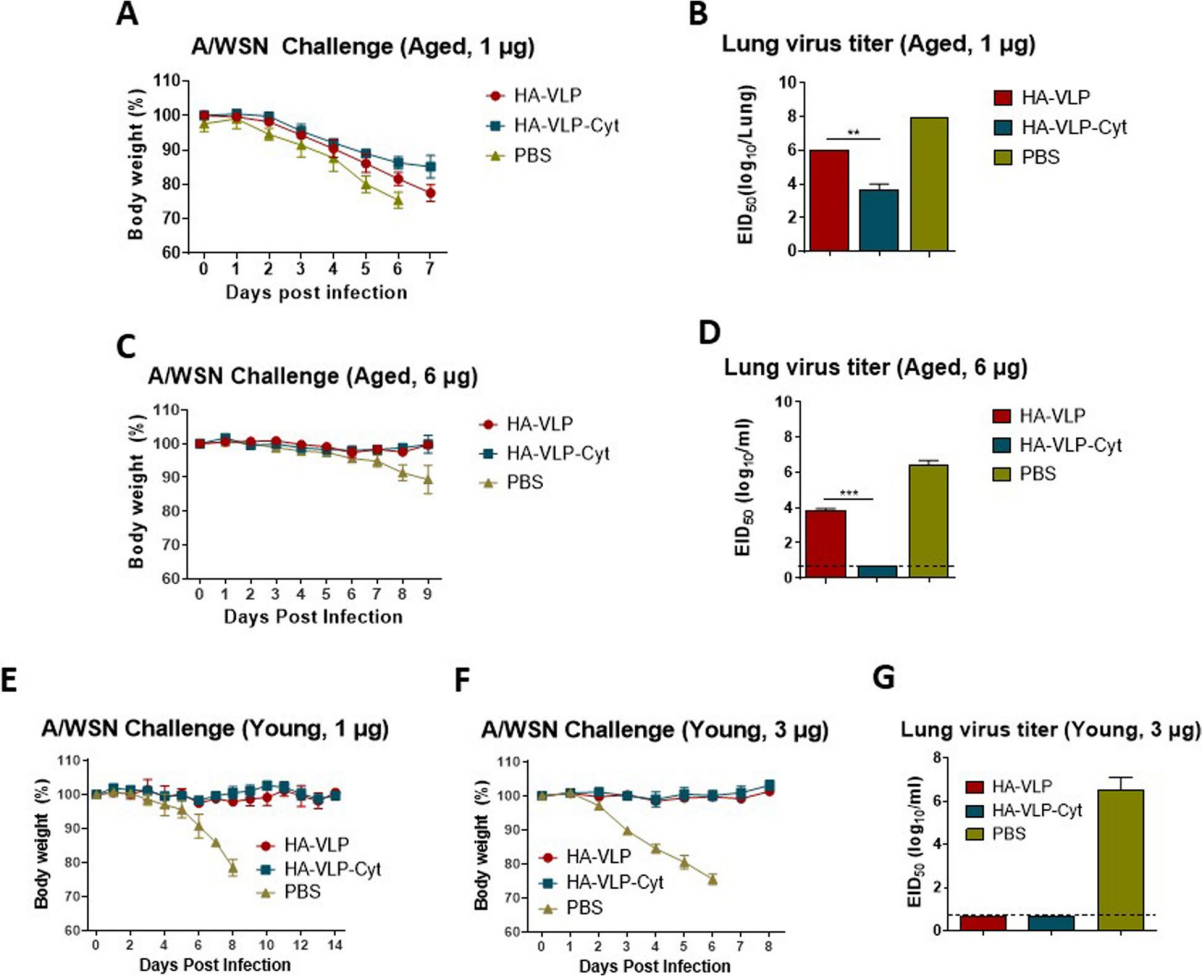
Higher dose of HA-VLP-Cyt vaccine reduces lung virus load in aged mice against heterologous virus. Aged BALB/c (*n=* 3-6/group) (**A**-**D**) mice received prime-boost immunization with 1 µg (**A**, **B**) or 6 µg (**C**, **D**) of HA-VLP or HA-VLP-Cyt before A/WSN infection. Young mice (**E**-**G**) prime-boost immunization with 1 µg (**E**) or 3 µg (**F**, **G**) of HA-VLP or HA-VLP-Cyt before A/WSN infection. Body weight changes (**A**, **C**, **E**, **F**) and lung virus titers at day 7 (**B**) or at day 8 (**D**, **G**) after A/WSN challenge (2 LD_50_, 11.6 EID_50_ for A, B, E, F, and G; 1 LD_50_, 5.8 EID_50_ for C and D). Error bars indicate mean ± SEM. The statistical significances were determined using One-way ANOVA and Turkey's multiple comparison test and indicated in ** *P <* 0.01; *** *P <* 0.001

To determine the effect of a higher dose of vaccine on heterologous cross-protection, aged mice were prime-boost immunized with 1 or 6 µg of HA-VLP or HA-VLP-Cyt and then challenged with A/WSN virus. Aged mice immunized with either 1 µg of HA-VLP or HA-VLP-Cyt showed body weight loss of 18% and 14%, respectively, after a lethal challenge (2 LD_50_) with A/WSN (Fig. 7A), while control (PBS) mice lost 25% of body weight after infection. However, HA-VLP-Cyt immunized mice displayed approximately 100-fold lower lung virus titer (3.7 log_10_EID_50_/ml) than HA-VLP group (6 log_10_EID_50_/ml) 7 days after infection. Both immunized groups showed significantly reduced lung virus titers compared to control group (8 log_10_EID_50_/ml) (Fig. 7B). To determine whether a higher dose of HA-VLP-Cyt vaccine confers better heterologous cross-protection in aged mice, we have challenged the mice with 1 LD_50_ dose of A/WSN virus. Since aged mice vaccinated with 6 µg HA-VLP or HA-VLP-Cyt maintained their body weight post infection with 1 LD_50_ dose (Fig. 7C), we determined lung viral titers. Approximately 300-fold lower lung virus titer was detected from the HA-VLP immunized mice (3.9 log_10_EID_50_/ml) than the control group (6.4 log_10_EID_50_/ml), whereas no virus was detectable in the HA-VLP-Cyt group (Fig. 7D).

To determine the cross-protective effect of the HA-VLP and HA-VLP-Cyt vaccination in young mice, young adult BALB/c mice were administered with 1 or 3 µg of  vaccine. At 3 weeks after HA-VLP prime and boost vaccination, mice were challenged with a lethal dose of heterologous A/WSN virus. HA-VLP or HA-VLP-Cyt vaccinated mice did not lose body weight after infection while the control group showed severe body weight loss and did not survive (Fig. 7E and F). The HA-VLP or HA-VLP-Cyt immunized mice were effectively cross-protected against A/WSN challenge, displaying no weight loss and lung virus titers below the limit of detection (Fig. 7G) on day 8 after challenge.

### HA-VLP-Cyt vaccine is more effective in inducing T cell responses than HA-VLP

**Fig. 8:**
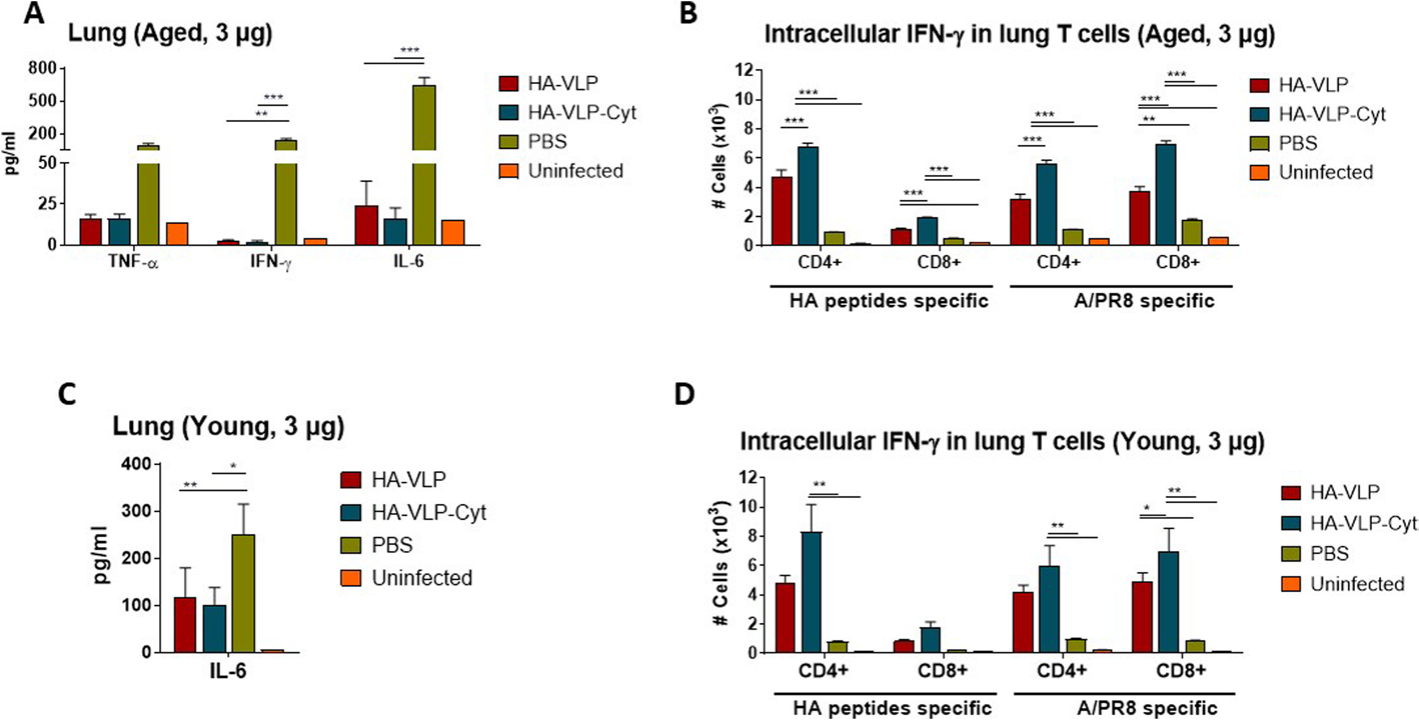
HA-VLP or HA-VLP-Cyt vaccination reduced inflammatory cytokines and induced T cell responses in the lung. Inflammatory cytokines and T cell responses were determined in lung tissues collected day 5 post challenge from aged BALB/c (**A**, **B**) and young (**C**, **D**) mice (*n=* 6/group) that were prime-boost immunized with 3 µg of HA-VLP or HA-VLP-Cyt. Inflammatory cytokines TNF-α, IFN-γ and IL-6 in lung lysates from aged mice after heterologous A/WSN challenge (**A**). IFN-γ-producing CD4^+^ T cells or CD8^+^ T cells specific for HA peptides and inactivated A/PR8 after intracellular staining were gated and quantified by flow cytometry from lung cells (**B**, **D**). Error bars indicate mean ± SEM. The statistical significances were determined using One-way ANOVA and Turkey's multiple comparison test and indicated in * *P <* 0.1; ** *P <* 0.01; *** *P <* 0.001

To determine whether the HA-VLP or HA-VLP-Cyt vaccination protected the mice by altering the local immune response in the lungs, lung tissue lysates were prepared and analyzed for inflammatory cytokines IL-6, TNF-α and IFN-γ by ELISA. Lung inflammatory cytokines (TNF-α, IFN-γ, IL-6) were significantly reduced in the vaccinated aged mouse groups with HA-VLP or HA-VLP-Cyt (3 µg) (Fig. 8A). A similar reduction of IL-6 cytokine in vaccinated young mice compared to naïve mice after A/WSN infection was observed (Fig. 8C). These data suggest that lung inflammation after infection can be prevented by prophylactic vaccination.

To determine the role of T cells in the protection conferred by HA-VLP vaccination, IFN-γ expression in CD4^+^ and CD8^+^ T cells in the lungs were analyzed by flow cytometry. Intracellular cytokine staining showed significantly increased levels of IFN-γ secreting CD4^+^ T cells and CD8^+^ T cells upon stimulation with HA peptides or inactivated A/PR8 virus in lung cells from HA-VLP or HA-VLP-Cyt (3 µg) vaccinated aged mice compared to unvaccinated naïve mice (Fig. 8B). Moreover, higher levels of IFN-γ secreting CD4^+^ T cells and CD8^+^ T cells (Fig. 8B) were observed in HA-VLP-Cyt boosted group compared to the HA-VLP vaccine group. Similar effects were observed in young adult mice (Fig. 8D). Taken together, these results indicate that HA-VLP-Cyt vaccination more effectively stimulates T cell responses in both young adult and aged mice than HA-VLP.

## Discussion

Vaccination is an effective measure to prevent infectious diseases, but vaccines are less effective in the elderly population compared to young adults [3]. This is mainly due to weakened immune system in the aged referred to as senescence [4, 6]. Several approaches in recent years include the use of high-dose vaccines [10, 11] and adjuvants to improve vaccine efficacy in the elderly [12]. To develop a more effective influenza vaccine for the elderly, we have utilized biological adjuvant incorporated HA-VLPs as a vaccine approach in aged mice. Prior clinical trials in healthy adults reported acceptable safety and immunogenicity of influenza HA-VLP vaccines (2009 H1N1, H5N1, H7N9) produced in insect cells [31–34], supporting HA-VLP as an acceptable vaccine platform. The efficacy of the HA-VLP vaccine in aged mice was markedly improved by the protein transfer-mediated incorporation of GPI-GM-CSF and GPI-IL-12 cytokine adjuvants. Protein transfer mediated incorporation of purified GPI-cytokine adjuvants onto HA-VLPs results in higher surface concentrations of cytokine adjuvants in comparison to techniques such as gene transfer [35, 36]. Moreover, protein transfer is a faster and highly efficient method compared to gene transfer techniques for incorporating GM-CSF into HA-VLPs [22, 37]. Our previous studies have shown that protein-transfer-mediated incorporation of GPI-GM-CSF resulted in up to 38.5% of total HA-VLP protein as compared to 0.1% incorporation by gene transfer techniques [22, 37]. GPI-anchored cytokines, when linked to particulate antigen source such as HA-VLPs, create a depot effect leading to slow release of cytokines at the vaccination site, thus reducing the systemic toxicity generally associated with soluble cytokines. In addition, HA-VLP-linked IL-12 and GM-CSF target APCs such as dendritic cells by binding to their receptors and enhancing antigen uptake and presentation [38–42], thereby enhancing T cell responses.

Although many adjuvants including MF59 were effective in improving the influenza vaccine efficacy, they are known to induce several side effects because of reactogenicity and toxicity [43]. Physiologically occurring biologic adjuvants offer several distinct advantages over chemically derived adjuvants (such as alum), TLR ligand-based adjuvants (such as CpG), or bacterial derived ones (such as MPL). Biologic cytokine-based adjuvants allow specific targeting to receptors on immune cells for antigen presentation, while cytokines such as IL-12 promote a Th1 type immune response. These qualities allow for a tailored immune response, which is critical in at-risk populations, such as the elderly, which are defective in their ability to generate and maintain protective immunity. In addition, GPI-anchored biologic adjuvants offer an excellent safety profile in comparison to traditional approved adjuvants, since they are physiologically occurring molecules and immobilized on a particulate HA-VLPs. This is critical when vaccinating elderly populations which require higher dose vaccines resulting in administration of increased amounts of adjuvants. Recent efforts in exploiting immunostimulatory cytokines as biological adjuvants to enhance efficacy of vaccines impacted the development of vaccines for children as well as adults. It is well documented that cytokines increase the efficacy of vaccines by attracting and activating key immune cells such as dendritic cells. IL-12 activates T lymphocytes and promotes development of a robust CTL response [44]. Pre-clinical and clinical trials performed to evaluate the potential of recombinant IL-12 as an adjuvant in treating several cancers and viral hepatitis resulted in enhanced immune response [45–49], but also resulted in unfavorable side effects and systemic toxicity [50, 51]. To circumvent the problems of limited immune response and toxic side effects, we engineered a membrane-associated GPI-anchored form of IL-12 which can be immobilized on amphiphilic particles to reduce the systemic toxicity. The GPI-anchor consists of a glycosylated moiety attached to phosphatidylinositol containing two fatty acids.

In addition to IL-12 we have also constructed a GPI-anchored form of GM-CSF, allowing targeting to dendritic cells to initiate an immune response. Blood monocytes derived from young (< 30 years) and aged (> 65 years) individuals differentiate into DCs in response to GM-CSF and IL-4 and produce similar amounts of inflammatory cytokines like IL-12 and TNF-alpha when stimulated with whole inactivated influenza virus [52], suggesting that aged DCs are as effective as young when induced by cytokines like GM-CSF and IL-4. It has been shown that GM-CSF as adjuvant in DNA vaccine promotes antibody avidity maturation [53] and cross-priming and increased cellular immune response via DC maturation [37, 39, 54–56]. We also found that using our protein transfer approach, only a low amount of GPI-GM-CSF (0.029 µg/ µg of HA-VLP) immobilized on the surface is needed for optimum antiviral response in mice, mitigating toxic side effects generally associated with soluble cytokines [22].

Our vaccine dramatically reduced lung viral titers in infected aged mice and protected them from both homologous and heterologous viral challenge. Highly effective viral clearance from the lung by several hundred-fold was observed in aged mice vaccinated with HA-VLP-Cyt (1 or 6 µg) compared to the control HA-VLP group. For the Fig. 7 heterologous A/WSN virus challenge, the weight loss pattern in the no vaccine PBS control mice was noted to differ between the Fig. 7A and C. This different pattern might be due to a different virus stock with lower infectious titers used for infection as reflected by little lower lung viral titers in Fig. 7C with 1 LD_50_ (5.8 EID_50_ units) compared to those in Fig. 7A with 2 LD_50_ (11.6 EID_50_ units) doses of A/WSN virus. Compared to the young adult mice (Fig. 7E), all aged mice even with HA-VLP or HA-VLP-Cyt displayed substantial weight loss of over 18% and 10% after 2 LD_50_ A/WSN virus (Fig. 7A-B). These outcomes suggest that aged mice appear to be more susceptible to A/WSN virus infection and show a delay in lung viral clearance than young adult mice. For this reason, in the other experiments, 1 LD_50_ dose of A/WSN virus was used to infect aged mice with 6 µg VLP vaccine dose, resulting in complete protection against weight loss compared to the PBS control (Fig. 7C-D). Nonetheless, we observed enhanced protective efficacy (1,000-fold reduction in viral titers) of HA-VLP-Cyt (6 µg dose) vaccination after heterologous A/WSN virus challenge compared to HA-VLP. Consistent with earlier studies [22], HA-VLP-Cyt was more effective in inducing IgG2a antibodies for heterologous A/WSN and CD4^+^ and CD8^+^ IFN-γ producing T cell responses than HA-VLP, suggesting that cytokines incorporated onto A/PR8 HA VLP exhibit adjuvant effects on enhancing cross-reactive IgG and Th1 type cellular immune responses. In the aged mice, HA-VLP-Cyt vaccination were more effective than HA-VLP in preventing weight loss against homologous A/PR8 virus challenge and in lowering lung viral titers after homologous (A/PR8) and heterologous (A/WSN) virus challenge. Induction of IFN-γ secreting CD4^+^ T cells was shown to contribute to host survival and recovery after infection [57], supporting a possible role of T cell immunity in cross-protection. In addition, HA stalk antibodies and cytotoxic CD8^+ ^T cells induced by HA-VLP vaccination could contribute to protection against influenza virus [58, 59]. Particularly, there have been several studies to develop universal influenza vaccines by targeting the conserved HA stalk domains [60–62]. However, HA stalk specific antibodies are not effectively induced by inactivated influenza virus vaccination [63]. We found that HA-VLP-Cyt vaccination was effective in inducing comparable levels of HA stalk specific IgG antibodies in young and aged mice. Thus, it is also possible that HA stalk specific IgG antibodies induced by HA-VLP-Cyt might have contributed to more effective cross-protection in aged mice compared to control HA-VLP.

## Conclusions

In summary, influenza HA-VLP vaccine adjuvanted with membrane-anchored biological adjuvants such as GPI-cytokines (HA-VLP-Cyt) enhanced protective immunity against both homologous and heterologous influenza A virus infection. Influenza HA-VLP-Cyt vaccine also induced enhanced T cell responses for better protection against heterologous infection in the absence of neutralizing antibodies. The results suggest cytokine-adjuvanted HA-VLPs could enhance protection against influenza A virus in the elderly who do not respond well to the currently approved vaccines. Taken altogether, a strategy of cytokine-adjuvanted HA-VLP vaccination might provide a safe and highly effective vaccine platform for the elderly against influenza viruses.

## Materials and methods

### Antibodies and proteins

Purified anti-mouse GM-CSF (clone MP1-22E9) and anti-mouse IL-12 (clone C17.8) were from BioXcell and used for affinity chromatography purification of GPI-GM-CSF and GPI-IL-12, respectively. GPI-GM-CSF and GPI-IL-12 were expressed in CHO-S cells and purified using affinity column chromatography as described in our earlier study [21].

### Preparation of influenza virus-like particles (HA-VLPs)

VLP expressing HA from A/Puerto Rico/8/1934 (A/PR8) were purchased from Medigen, Inc. (Frederick, MD). In brief, codon-optimized HA gene was cloned into pFastbac plasmid vector with influenza M1 gene for dual expression (M1 + HA). Recombinant baculovirus (rBV) expressing M1 and HA was generated in insect cells by using the Bac-to-Bac expression system and transfection with M1 + HA pFastbac bacmid DNA as previously described [63–65]. VLPs were produced in Sf9 insect cells after infection with rBV expressing HA and M1. Soluble cytokines were expressed and purified from CHO-S cells after transfection with GM-CSF or IL-12 expressing vectors including the glycosylphosphatidylinositol (GPI)-membrane-anchoring sequence derived from CD59. Purified GPI-cytokines (GPI-GM-CSF and GPI-IL-12) were incorporated onto HA-VLP by protein transfer (HA-VLP-Cyt) as previously described [21]. Briefly, 1 mg HA-VLP was incubated with 25 µg of GPI-GM-CSF and GPI-IL-12 molecules were mixed and incubated at 37 °C for 3 h with constant rotating in an incubator. Unincorporated GPI-GM-CSF and GPI-IL-12 were removed by centrifugation at high speed in an ultracentrifuge for 1 h. Characterization of HA-VLP was performed by Coomassie blue staining of sodium dodecyl sulfate polyacrylamide gel electrophoresis (SDS-PAGE) and incorporation of GM-CSF or IL-12 was determined by western blot analysis using anti-mouse GM-CSF (Clone MP1-22E9) or anti-mouse IL-12 p40 (Clone C17.8) antibodies (BioXCell, Lebanon, NH) respectively. Western blots were revealed using the secondary antibody AP Goat anti-rat IgG (Jackson Immunoresearch Laboratories, West Grove, PA) and NBT/BCIP as a substrate (ThermoFisher Scientific, Waltham, MA). GPI-cytokine incorporation into HA-VLP was also confirmed by flow cytometry analysis using APC anti-mouse GM-CSF (clone MP1-22E9, BioLegend, San Diego, CA) and PE anti-mouse IL-12 p40 (eBioscience/ThermoFisher Scientific, Waltham, MA). Samples were run on a BD FACSCalibur and analyzed using FlowJo software (FlowJo V10, TreeStar Inc, BD Biosciences).

### Immunization and viral challenge studies

Young adult (6–8 weeks old, Jackson Laboratories) and aged female (18-month-old, NIH/NIA) BALB/c mice received sequential prime-boost intramuscular vaccination with HA-VLP or HA-VLP-Cyt (HA-VLP with GM-CSF and IL-12 incorporated) at an interval of 3 weeks. Before homologous virus challenge, mice (*n* = 6/group) were prime-boost immunized with 1 µg, 3 µg, or 6 µg of HA-VLP or HA-VLP-Cyt. Challenge viruses include homologous A/PR8/34 and heterologous A/WSN/1933 (A/WSN/33) H1N1 viruses. A/PR8/1934 and A/WSN/1933 viruses were kindly provided by Dr. Huan Nguyen (University of Alabama at Birmingham, AL) and by Dr. Yumiko Matsuoka (CDC, Atlanta, GA) as previously described [64]. These viruses were amplified in chicken eggs after inoculation with 1 hemagglutination activity unit as previously described [65]. For infecting mice, different stocks of A/WSN virus with different levels of infectivity (1 LD_50_ and 2 LD_50_) were used in each experiment of aged mice that was vaccinated with 1 or 6 µg VLP, respectively. The doses of challenge viruses were determined by 50% lethality in mice (LD_50_), which were presented in egg infectious dose (EID_50_) units retrospectively based on EID_50_ units of the virus stocks. The LD_50_ of virus was determined in BALB/c mice (6–8 weeks old) after intranasal inoculation with 50 µl of 10 folds serial dilutions of each stock of A/PR8/1934 and A/WSN/1933 viruses every time when new stocks were prepared. EID_50_ were determined after inoculation with 200 µl of 10 folds serial dilutions of each virus stock. The positive infection was identified by hemagglutination activity assays of allantoic fluids harvested from all eggs inoculated with 10 folds serial dilutions of each virus stock. The mice displaying weight loss (over 20%) were humanely euthanized to avoid severe painful illness. Survival rates were presented, based on the endpoint of 20% weight loss. All animal experiments were approved by the Georgia State University Institutional Animal Care and Use Committee (IACUC). Mouse animal experiments including virus infection, blood and tissue collections were performed in accordance with the approved IACUC protocol (A21004) and regulations.

### Enzyme linked immunosorbent assay (ELISA)

IgG antibodies specific for the viruses were measured by ELISA with coating antigens (4 µg/ml) from inactivated A/PR8/34, A/WSN/33, or A/California/04/2009 (A/Cal/09) H1N1 viruses. Antigen-specific IgG concentrations are quantified using a standard curve for IgG antibody (SouthernBiotech). A/PR8/34 specific IgG isotypes (IgG1 and IgG2a) were measured. HA stalk specific IgG antibodies were determined using consensus group 1 HA stalk protein as coating antigens constructed and prepared as previously reported [66]. Horseradish Peroxidase (HRP) conjugated goat anti-mouse IgG, IgG1, and IgG2a (SouthernBiotech) were used as a secondary antibody. 3,3′,5,5′-Tetramethylbenzidine (TMB) substrate (Invitrogen™) was utilized for color development. Absorbance was read at 450 nm by BioTek ELISA plate reader.

### Hemagglutination inhibition (HAI) titers

Mouse prime-boost immunized sera were treated with receptor destroying enzymes (RDE, Sigma) with 1:3 ratio and incubated at 37°C overnight and inactivated at 56°C for 30 min. RDE-treated serum samples were serially diluted (two-fold) and treated with the equal volume of viruses (4 hemagglutination activity units). HAI titers were measured as the highest dilution factor inhibiting the formation of buttons with 0.5% chicken red blood cells (RBC).

### Assays of lung viral titers and inflammatory cytokines

Due to slow viral clearance in aged mice, lung lysates were obtained at different time points, day 6–8 post infection from the lung extracts in 1.5 ml of RPMI media. Virus titers were determined by calculating 50% egg infectious dose (EID_50_) after incubation at 37ºC for 3 days in the 10-day-old embryonated chicken eggs. Interleukin 6 (IL-6), tumor necrosis factor alpha (TNF-α), and interferon gamma (IFN-γ) ELISA was performed using Ready-Set-Go kits (eBioscience, San Diego, CA).

### Intracellular cytokine staining of T cells

For T cell immune responses primed by vaccination, lung samples were collected early at day 5 post infection to minimize the induction of adaptive immune responses in control unvaccinated mice. Lung cells were stimulated with inactivated A/PR8/33 (4 µg/ml) or A/PR8 HA peptides (CD4^+^ T cell epitopes (2.5 µg/ml each peptide): SFERFEIFPKE, HNTNGVTAACSH, CPKYVRSAKLRM, KLKNSYVNKKGK, NAYVSVVTSNYNRRF, CD8^+^ T cell epitopes (5 µg/ml each peptide): LYEKVKSQL, IYSTVASSL for 5 h at 37 °C in the presence of brefeldin A. After stimulation, lymphocytes were stained with T cell marker antibodies for CD4 (CD4-PE/Cy5, BD Biosciences) and CD8 (CD8α-PE, Biolegend) and then stained for cytokines according to manufacturer’s protocol from BD Cytofix/Cytoperm Plus Kit. Intracellular staining of the permeabilized lymphocytes was conducted with IFN-γ cytokine mAb (anti-mouse IFN-γ-APC/Cy7, BD Biosciences). Cells stained were acquired by using LSR-II/Fortessa flow cytometer (BD Biosciences, San Diego, CA, USA) and analyzed using the FlowJo software (FlowJo V10, Tree Star, Inc., BD Biosciences).

### Statistical analysis

Two-way or one-way ANOVA were used to determine the statistical significance when comparing two different conditions. P-values of less than or equal to 0.05 were considered significant. Data analysis was performed using Prism software (GraphPad software Inc., San Diego, CA).

## Data Availability

All data are available in the main text.
